# Impulsivity across reactive, proactive and cognitive domains in Parkinson's disease on dopaminergic medication: Evidence for multiple domain impairment

**DOI:** 10.1371/journal.pone.0210880

**Published:** 2019-02-13

**Authors:** Nádia Canário, Mário Sousa, Fradique Moreira, Isabel Catarina Duarte, Francisco Oliveira, Cristina Januário, Miguel Castelo-Branco

**Affiliations:** 1 Coimbra Institute for Biomedical Imaging and Translational Research (CiBit), ICNAS—Institute for Nuclear Sciences Applied to Health, Brain Imaging Network of Portugal, Coimbra, Portugal; 2 Laboratory of Biostatistics and Medical Informatics, Institute for Biomedical Imaging and Life Sciences (CNC.IBILI), Faculty of Medicine, University of Coimbra, Coimbra, Portugal; 3 Division of Movement Disorders, Department of Neurology, Coimbra Hospital and University Centre, Coimbra, Portugal; University of Queensland, AUSTRALIA

## Abstract

Impulse control disorders (ICD) may occur in Parkinson’s disease (PD) although it remains to be understood if such deficits may occur even in the absence of a formal ICD diagnosis. Moreover, studies addressing simultaneously distinct neurobehavioral domains, such as cognitive, proactive and reactive motor impulsivity, are still lacking. Here, we aimed to investigate if reactive, proactive and cognitive impulsivity involving risk taking are concomitantly affected in medicated PD patients, and whether deficits were dependent on response strategies, such as speed accuracy tradeoffs, or the proportion of omission vs. commission errors. We assessed three different impulsivity domains in a sample of 21 PD patients and 13 matched controls. We found impaired impulsivity in both reactive (p = 0.042) and cognitive domains (p = 0.015) for the PD patients, irrespective of response strategy. For the latter, effect sizes were larger for the actions related with reward processing (p = 0.017, d_Cohen_ = 0.9). In the proactive impulsivity task, PD patients showed significantly increased number of omissions (p = 0.041), a response strategy which was associated with preserved number of commission errors. Moreover, the number of premature and proactive response errors were correlated with disease stage. Our findings suggest that PD ON medication is characterized compared to healthy controls by impairment across several impulsivity domains, which is moderated in the proactive domain by the response strategy.

## Introduction

Parkinson's disease (PD) is a progressive neurological disorder in which the loss of dopaminergic nigrostriatal neurons, alongside with associated secondary dysfunctions in cholinergic, noradrenergic and serotonergic neurotransmitters, leading to motor, cognitive and behavioral dysfunctions [1,2]. Concerning impairments in the behavioral domain, evidence suggests a diminished capacity in the execution of inhibitory programs leading to a variety of impulsive behaviors [3,4] even in the absence of an overt impulse control disorder [5]. In line with the idea that this cognitive domain may be affected early on in neuropsychiatric disorders, we also previously found impaired response inhibition in a neurodevelopmental disorder, Neurofibromatosis Type 1, even in the absence of clinical criteria for Attention Deficit and Hyperactivity Disorder (ADHD) [6].

Although impulsivity is a broad psychological construct that can be divided in different domains [7], it can overall be defined as a tendency to act prematurely without forethought or planning [8]. The International Classification of Diseases -10 (ICD-10) describes impulsivity as “repeated acts that have no clear rational motivation and that generally harm the patient's own interests and those of other people” (p.165) [9]. According to Bechara, Damasio and Damasio [10] one can divide impulsivity in two separable domains: cognitive impulsivity and motor impulsivity. Cognitive impulsivity relates to the inability to inhibit cognitive demands leading to immediate gratification [10]. This type of impulsivity can be expressed in impaired decision making [11], and it is frequently involved in risk taking activities, such as gambling, drug-seeking and several other aspects of addictive behaviors [8,11]. On the other hand, motor impulsivity refers to the impaired inhibition of a previously learned motor response, which can be divided in reactive and proactive impulsivity [11]. The former refers to the inability to inhibit a motor program in the presence of a specific stop-signal, where the need to stop is considered to be irrevocable [11,12]. Proactive impulsivity relates to an impairment in withholding a response, where the stop-signal is motivated by external contextual cues, which helps anticipating the inhibitory program. It is therefore more goal-directed, selective and triggered by environmental cues [11–14].

Dopamine replacement therapy (DRT), which includes the intake of a dopamine precursor–levodopa -, and dopamine agonists (DA) [15] has been identified as potentially contributing to both cognitive, and motor impulsivity. Regarding the first, behaviors like binge eating, compulsive shopping, and gambling [16–18] have been suggested to represent evidence of disrupted cognitive control. We previously identified an important role for a dichotomy within corticostriatal structures underlying the imbalance in habitual versus goal directed neural actions [19], which might be relevant for all above mentioned impulsivity domains. Since these circuits are affected in PD, it is relevant to study all these impulsivity domains concomitantly in the same patients. Using risk-taking measures, decreased performance was observed in medicated PD patients, which tend to show a lower sensitivity to punishment [20–26]. The presence of disrupted reactive inhibitory control is consistent with reduced performance in go/nogo tasks in medicated PD when compared with normal subjects [27,28]. Likewise, impaired inhibition in tasks requiring context guided response control is also recognized [27,28], whose deficits might stem from dysfunction in cortico-basal ganglia loops [11,24,29,30]. Previous human studies attempted to unravel the behavioral effects of dopamine replacement drugs in the cognitive impulsivity domain [3,27,30,31,33]. A few studies addressed simple reactive [32–34] or proactive paradigms [27,28]. The number of studies dedicated to these last domains is relatively scarce.

To the best of our knowledge, no study has so far simultaneously analyzed all the aforementioned impulsive behavior, which is important for a comprehensive understanding of impulsivity profiles in this disease. Thus, the present study aimed to address this issue by simultaneously investigating 3 different domains of impulsive behavior (reactive, proactive and cognitive) in medicated PD, as compared with healthy controls.

## Materials and methods

### Participants

We initially assessed 48 PD patients with idiopathic PD from the Movement Disorders Unit of the Coimbra University Hospital. Given our stringent exclusion criteria, and to prevent confounding effects, 27 PD patients (~56%) from this total sample were further excluded: 26 patients were excluded due to high scores on Beck Depression Inventory –2 (BDI-2, > 20 points) and 1 patient was excluded due to MoCA score below the normative value expected for this participant, according to age and education. In the final tested sample, we examined the multidomain impulsivity profile in 21 medicated patients with idiopathic PD and 13 healthy controls, matched for age (PD mean ± SD, 69.99 ± 8.12 / Controls mean ± SD, 66.23 ± 7.18, t(32) = 1.009, p = 0.321) and level of education (PD mean ± SD, 4.71 ± 2.43 / Controls mean ± SD, 4.85 ± 2.30, U = 125.50, p = 0.639). Patients were enrolled between December, 2014 and June, 2015. All PD patients were on a stable DRT at least for 3 months. Five patients were also taking antidepressant agents and none of the patients were taking neuroleptic agents. PD patients submitted to Deep Brain Stimulation surgery or infusion therapies were not included in this study. In Table 1, we present the complete demographic, clinical and neuropsychological data of all participants. We reported DRT as Levodopa equivalent daily doses (LEDd) according to reported elsewhere [35]. See supporting information (S1 Table) for detailed clinical information of all patients.

**Table 1:** Demographic, clinical and neuropsychological characteristics of participant groups.

PD		Controls
**Age (mean, sd)**	69.00 (8.12)	66.23 (7.18)
**Gender (F:M)**	12:9	8:5
**Education (mean, sd)**	4.71 (2.43)	4.85 (2.30)
**Disease duration (mean, sd)**	7.14 (5.31)	-
**H&Y (mean, sd)**	2.29 (0.41)	-
**UPDRS-III ON (mean, sd)**	24.86 (8.49)	-
**UPDRS-III OFF (mean, sd)**	41.19 (9.10)	-
**LEDd total (mean, sd)**	743.38 (455.12)	-
**LEDd DA (mean, sd)**	291.91 (241.41)	-
**BDI-2 (mean, sd)**	9.57 (4.71)	5.69 (2.69)
**AES (mean, sd)**	25.43 (5.34)	21.46 (3.93)
**MoCA (mean, sd)**	22.67 (2.99)	23.46 (2.99)

Legend: sd = standard deviation; H&Y = Hoehn & Yahr; MDS-UPDRS = Movement Disorder Society-Unified Parkinson's disease rating scale; LEDd = Levodopa Equivalent Dose; BDI-2 = Beck Depression Inventory-2; AES = Apathy Evaluation Scale; MoCA = Montreal Cognitive Assessment.

The study was approved by the ethics committee of the Faculty of Medicine of the University of Coimbra. Written informed consent was obtained from all participants.

Exclusion criteria for this study were: a) previous diagnosis of psychiatric disorders; b) presence of significant depressive symptomatology; c) presence of apathy, and d) cognitive impairment. The presence of depressive symptomatology was accessed by the self-rated BDI-2 [36,37]. Considering the presence of a somatic factor on BDI –2, which easily increases the total value on this measure leading to false positives, the cut-off point was adjusted to 20, for the patients, excluding cases of moderate and severe depression. Apathy symptoms were assessed by Portuguese version of the Apathy Evaluation Scale (self-rated version) [38] and cognitive impairment was excluded using the Portuguese validation of the Montreal Cognitive assessment (MoCA) [39]. All the assessments related to the exclusion criteria were done during the “on” medication state. The diagnosis of idiopathic PD was performed by a neurologist specialized in movement disorders according to the UK Brain Bank Criteria for Parkinson’s Disease [40]. Motor severity was assessed using the motor subscore (part III) of the Movement Disorders Society Unified Parkinson's Disease Rating Scale (MDS-UPDRS-III), measured both in “on” and “off” medication state. All participants had normal or corrected-to-normal vision and gave their informed written consent for the study according to the Declaration of Helsinki.

### Materials

#### Go/nogo task

This task, which we used before to study response inhibition in Neurofibromatosis type I [6] was inspired by the sustained attention to respond task (SART) [41] and was included here in order to study the (motor) reactive impulsivity domain. In this task subjects viewed white single digits from 1 to 9 (Helvetica font) randomly presented. Digits were displayed in a black background at the center of the computer screen for 250 ms with an inter-trial interval of 1500 ms. Each digit was followed by a white fixation cross until the next digit appeared. Digits size varied randomly between 5 different sizes {48, 72, 94, 100 and 120}. Participants were asked to press a button with their dominant hand every time they saw any number (1, 2; 4–9, go stimuli), except for the number “3” (nogo stimulus). The experiment was divided in 2 repeated blocks. Each block had 225 trials, including 25 nogo trials (11%) and 200 go trials, and lasted for 5:24 minutes (overall time of 10:48 min.) The go/nogo task was presented using the Psychophysics Toolbox [42] for Matlab R2014a (MathWorks, Natick, USA).

#### AX Continuous Performance Task (AX—CPT)

This task was inspired by Rush and colleagues [14] and allows to analyze both reactive and proactive domains of impulsivity. In this task participants saw at the center of the screen, and one at the time, the letters A, X, B and Y, in the following possible order: AX, AY, BX and BY. Letters were designed in white color and displayed on a black background. Like the previous experiment, letters size varied pseudo randomly between 5 different sizes {48, 72, 94, 100 and 120}. Each letter was presented on the screen for 250 ms and was followed by a 1000 ms interstimulus interval. There were a total of 140 AX trials (70%), 20 BX trials (10%), 20 AY trials (10%) and 20 BY trials (10%). Thus AX trials were the go trials, whereas BX, AY and BY were the nogo trials. Subjects were instructed to press the spacebar every time an “X” appeared immediately after an “A” cue. On the contrary, whenever they saw a “B” they were told to not respond to the trial regardless of the letter presented immediately after. The BX trial was introduced as one of the way to analyze the proactive control, since the contextual presence of the nogo-cue -“B”- prepares the participants to stop a previous response [13]. On the other hand, the AY trials, represent a correct cue and an incorrect probe, where “A” establishes an expectancy to make a response to an expected probe. Finally, BY trials (representing an incorrect cue and an incorrect probe) were designed to be control trials where there was no response competition. These trials were used as a direct exclusion criteria for the AX-CPT task, since if the subjects made at least 50% or more BY errors they would be excluded from the task, based on the possibility that they might not have understood the task demands. All trials were presented in a randomized order. The present experiment was divided in 2 repeated blocks, each of one lasting for 8:35 minutes (overall time of 17:10 min.). The AX-CPT task was presented using the 4.4 version of SuperLab (Cedrus Corp., San Pedro, CA).

#### Ballon Analogue Risk Taking (BART) task

This task was inspired by Lejuez et. al [43] and was included in order to study the cognitive impulsivity domain. It consists in a measure of risk taking propensity. During this task subjects begin to see a small blue balloon at the center of the computer screen accompanied by the display of the cumulative amount of money earned at their left–“*Total*”-, and by the amount of money earned in the present trial–“*X euros*”-, at their right. Subjects were told: “You are going to see a total of 30 balloons, one at each time, and your job is to pump each balloon pressing the spacebar button. For every pump the balloon will be filled a little bit more. But remember, balloons would explode if they were pumped past its explosion point. The decision to stop is up to you. Some of these balloons will explode after few pumps while others will only explode after they almost filled the computer screen. For every pump you will earn 1 cent, but if the balloon explodes you will lose all the money you collected in this trial. In order to collect the money earned with each balloon, stop pumping and press the “C” button and this value will be transferred to your wallet. Every time you collect money or a balloon explodes, a new balloon will appear”. All balloons had a different unpredictable “explosion” point with the smallest balloon exploding on the 5^th^ pump and the largest exploding after 61 pumps. Subjects could earn a maximum of 9.33 euros and all of them received the money after completing the task, at the end of the session. All trials were presented in a randomized order. The Bart task was presented using the toolkit Vizard 3.0 (WorldViz. Santa Barbara. USA).

**Fig 1:**
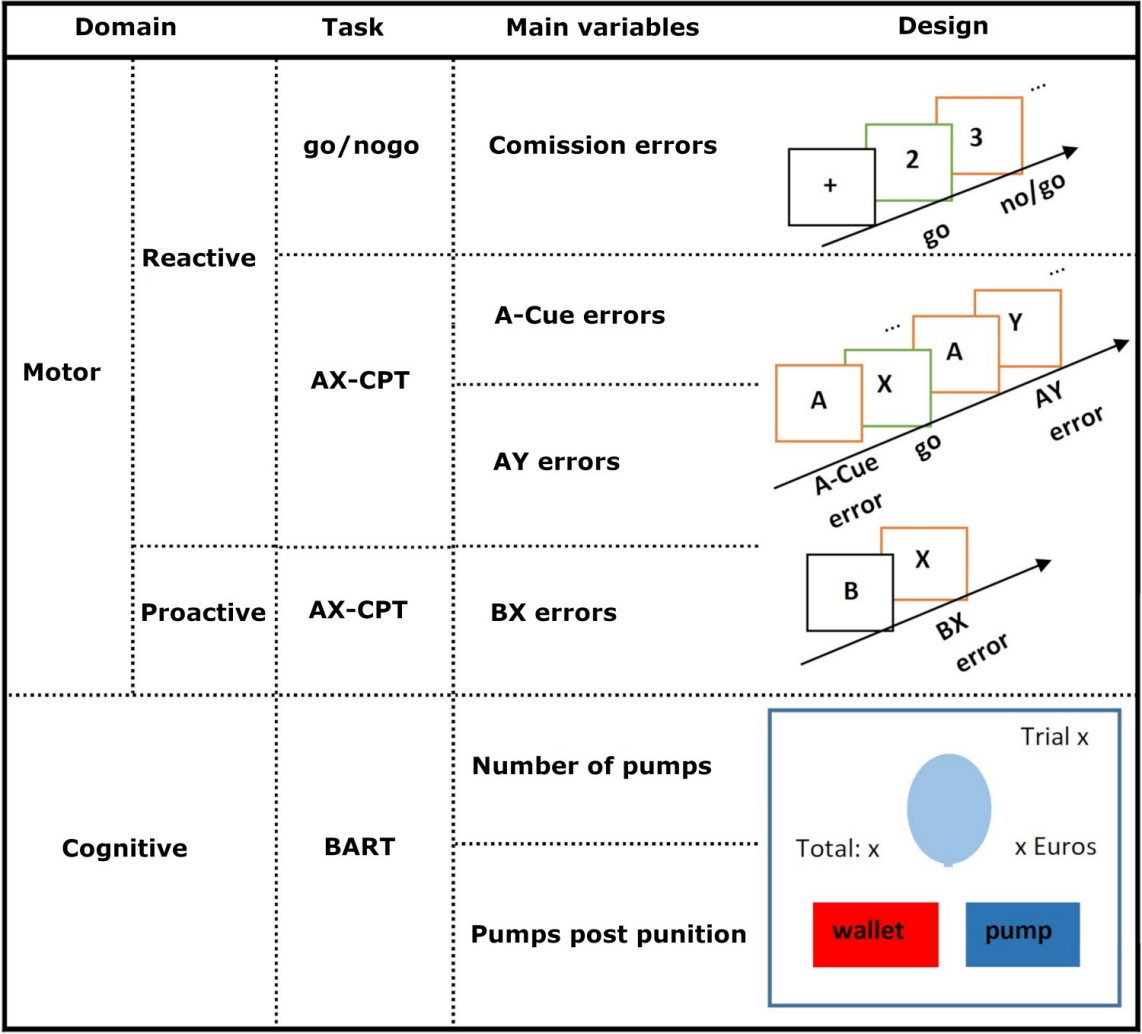
Summary of experimental tasks.

A summary of all investigated impulsivity domains and their respective tasks and the means of their evaluation are summarized in Fig 1.

### Procedures

PD patients performed the go/nogo task, AX-CPT and BART during “on” medication state, in a counterbalanced order for the presentation of tasks. “On” state was defined as 60–150 min after the intake of DRT (in some patients slightly time adjustments were made, by the clinician, to guarantee that patients were in the “best on” state). The experimental procedures always started with the application of the MDS-UPDRS motor score, and followed by one of the three experimental tasks. The experimental tasks were presented in a portable computer (ACER ASPIRE, CPU INTEL^R^, CORE ^TM^i7).

### Data analysis

#### Go/nogo

The main dependent measure from the go/nogo task was the number of commission errors (CE), which represent the failures to withhold the motor response for nogo trials. We also recorded the mean total number of omissions and the overall reaction time (RT). Independent sample t tests were performed for PD vs controls in order to compare CE. Omissions and RT´s were independently analyzed. We also calculated Pearson’s correlations between the CE and the RT’s, to evaluate speed accuracy trade-offs, followed by a linear regression analysis.

#### AX-CPT

Main dependent measures in this task were: AY errors, BX errors, and A-Cue errors. The A-Cue errors shared the same principle as the AY errors since the (go) cue increased the likelihood for pre-activation of a “go” response. However, this susceptibility to enhanced pre-activation condition could be more pronounced, given that participants might not wait for the next trial, giving a response during the presentation of the cue. Like the former task, we also recorded the mean total omissions and the overall RT. The performance of 2 participants was excluded from the analysis. The exclusions were due to struggle in understanding the tasks demands (PD group, 1 taking levodopa, and 1 also taking DA in addition). Nonparametric one-way MANOVAs were performed for the PD vs controls regarding the AY, BX, A-Cue errors. P-values extracted from the MANOVA are already corrected for multiple comparisons in which concerns the multiple response outcomes (see [44]). Indeed, when more than one dependent variable is measured, MANOVA corrects for the number of these dependent variables. Like in the go/nogo task the number of omissions and the RT´s were also independently analyzed. Also for the AX-CPT task we performed a Pearson’s correlation between each type of error—AY, BX and A-Cue—and the RT’s followed by a linear regression analysis.

#### BART

Main dependent measures from the BART were the number of adjusted pumps (the average number of pumps actions)—higher values would reflect a greater risk tasking -, and the mean number of pumps post punition (the average number of pumps without exploding the balloon, computed after a trial where the balloon previously exploded). The number of pumps post punition was taken in order to study the sensitivity towards punition. Accordingly, the statistical analysis regarding these variables was performed using MANOVA, using age and education as covariates for the particular comparison between the two PD groups. Behavioral data were analyzed using the IBM SPSS statistical package (v.22). An alpha level of 0.05 was used for all statistical tests.

Moreover, for all main dependent variables we also computed an exploratory correlation analysis between those and the disease stage measured by the Hoehn & Yahr (H&Y) scale and between these behavioral measures and the total LEDd and LEDd for agonist medication. Given that this part of the analysis is exploratory instead of confirmatory, interpretation of uncorrected p values in this case is just hypothesis generating [45].

Given the difficulty in finding age-matched controls we also pre-calculated a minimum sample size needed for the control group, for a ratio 2/1, for the mean number of pumps from the BART. The analysis suggested that with an anticipated mean of 19 number of pumps for the PD group and of 12 number of pumps for the control group and with a standard deviation of 6, and with an expected power of 80% and an alpha level of 5%, our sample size for the control group should at least contain 9 participants (and 18 for PD).

## Results

**Fig 2:**
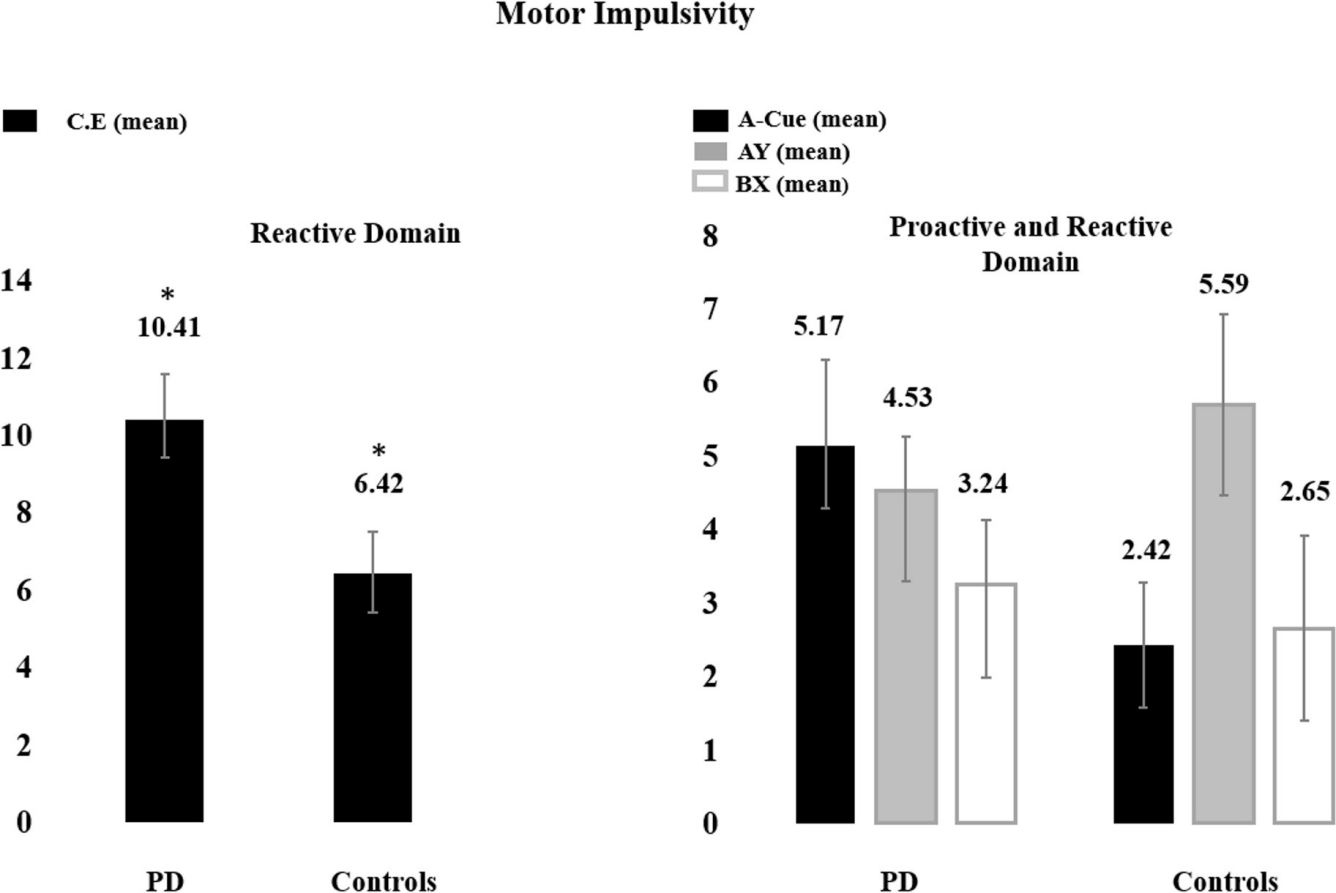
Bar plots depicting mean and standard error of the mean for main dependent variables in the motor impulsivity tasks–go/nogo and AX-CPT tasks. The Figure depicts both performance for PD´s and controls. * depicts the significant differences at p < 0.05. C.E.–Commission Errors.

**Fig 3:**
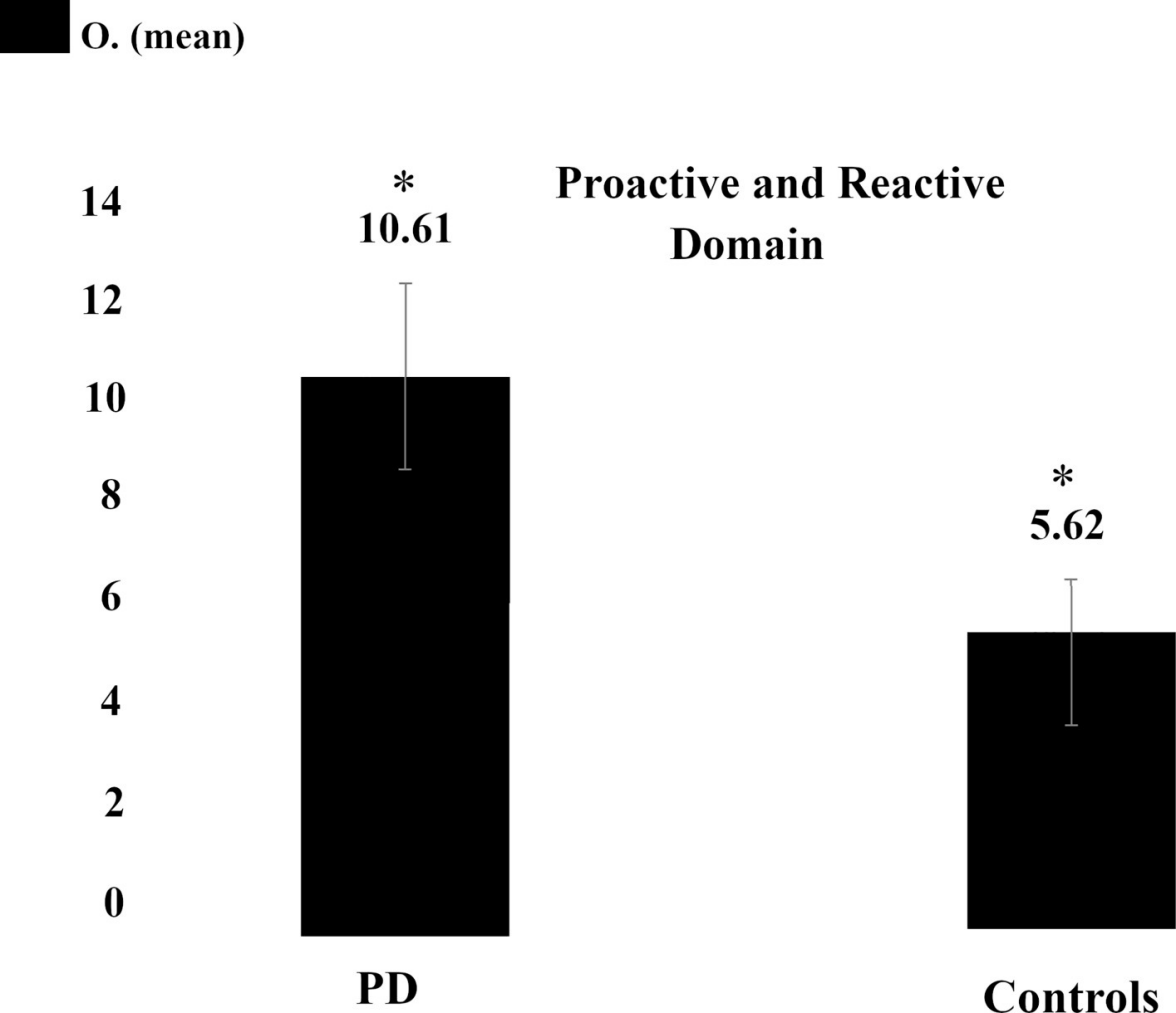
Bar plots depicting mean and standard error of the mean for the omissions in AX-CPT task. Figure depicts both performance for PD´s and controls. * depicts the significant differences at < 0.05. O.–Omissions.

**Fig 4:**
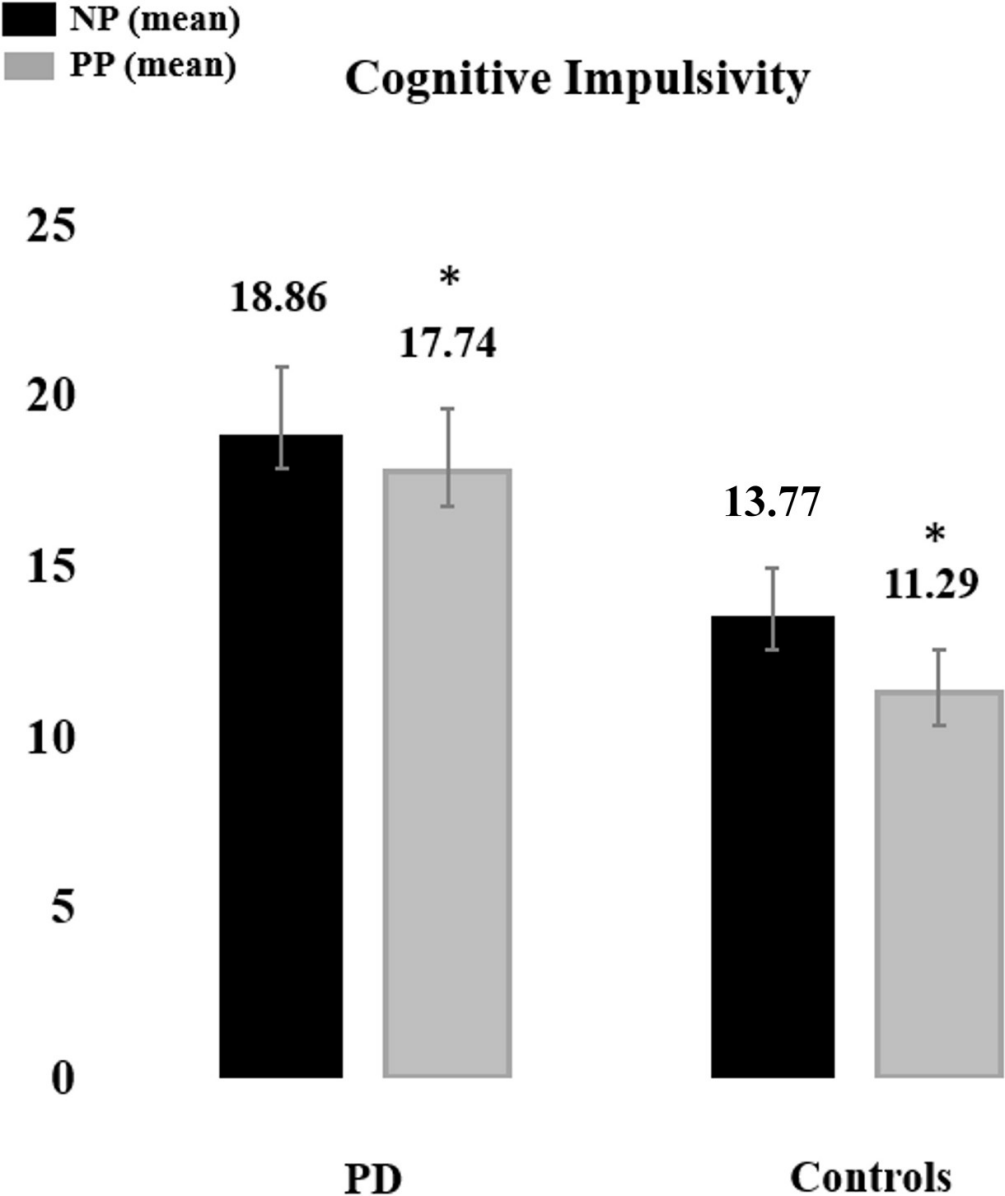
Bar plots depicting mean and standard error of the mean for main dependent variables in the cognitive impulsivity task–BART task. Figure depicts both performance for PD´s and controls. * depicts the significant differences at < 0.05. C.E.–Commission Errors; NP–Number of Pumps; PP–Pumps Post Punition.

Mean and standard errors for the main dependent variables on the three tasks are depicted in Figs 2, 3 and 4. Significant differences between controls and patients were observed in the go/nogo task (t (31.765) = 2.117, p = 0.042, d_Cohen_ = 0.1), with the latter showing a higher number of CE for the PD group (mean ± SD, 10,41 ± 7.04), when compared to healthy controls (mean ± SD, 6,42 ± 3,92). Concerning cognitive impulsivity, we found significant multivariate (MANOVA) effect for the variables pumps post punition and number of pumps concerning both groups (Pillais’ trace = 0.238, F (2.31) = 4.846, p = 0.015). Post-hoc analysis investigated the sources of this effect. This analysis for the effect of the number of pumps post punition showed that PD patients committed a higher number of pumps post punition (mean ± SD, 17,74 ± 8,57) compared to controls (mean ± SD, 11,29 ± 4,45) (F (1) = 6.284, p = 0,017, d_Cohen_ = 0.9).

Regarding the AX-CPT task, we did not find a multivariate (non-parametric MANOVA) effect for the three main dependent variables (A-Cue, AY and BX) (*X*^2^ (3) = 6.324, p = 0.09), which might be explained by an effect of response strategy (see below). Nevertheless, individual analysis of AX-CPT errors revealed a significant between group difference in A-Cue errors (U = 69.500, p = 0.037). Analysis for the omissions in this task showed that the PD patients made more omissions (mean ± SD, 10,61 ± 7.68), comparing to the control group (mean ± SD, 5.62 ± 4.08; t (30) = 2.137, p = 0.041, d_Cohen_ = 0.8), suggesting a strategy to increase omission as a way to minimize commission errors, which thereby become masked (Fig 3). No differences were found in the RT’s in any of the tasks.

**Fig 5:**
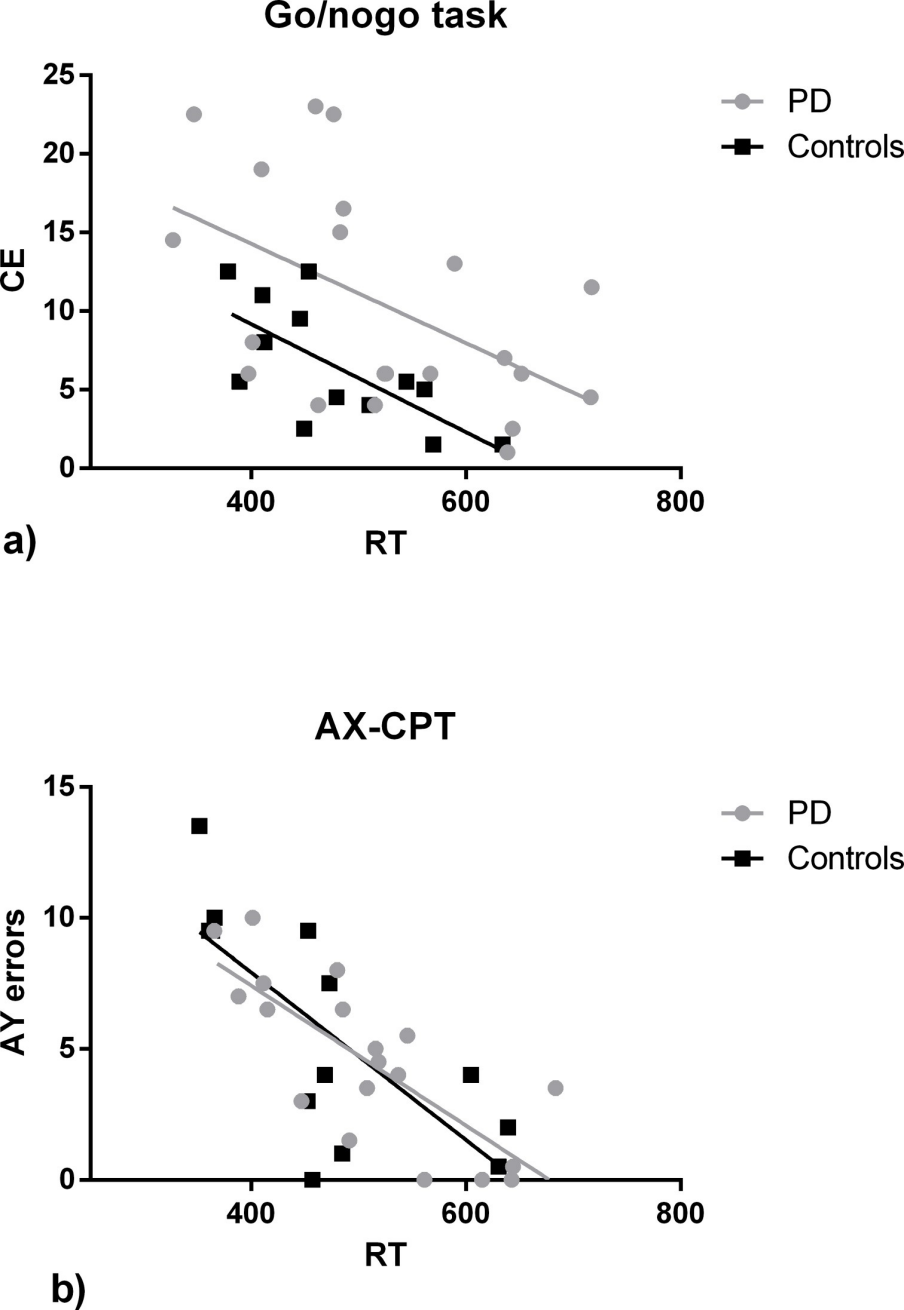
**a)** Regression plots depicting mean CE of each PD patient and control vs. RT for the go/nogo task. **b)** Regression plots depicting mean AY errors of each PD patient and control vs. RT for the AX-CPT task. Solid lines represent the regression curves. Regression curves for the go/nogo task: PD, R^2^ = 0.2659, Y = -0.03156*X + 26.89; Controls, R^2^ = 0.4769, Y = -0.03436*X + 22.90. Regression curves for the AX-CPT task: PD, R^2^ = 0.5999, Y = -0.02668*X + 18.08; Controls, R^2^ = 0.5296, Y = -0.03193*X + 20.68. C.E.–Commission Errors.

Regarding of response strategy and speed accuracy tradeoffs analysis, correlation analysis across groups revealed a similar speed accuracy tradeoff (SAT) for both CE from the Go/nogo task and the AY errors from the AX-CPT, corroborating the notion that response strategies were similar concerning speed accuracy tradeoffs. Indeed, we found a moderate negative correlation between CE and RT’s for both groups (PD: r = - 0.516, p = 0.017, N = 21; Controls: r = -0.691, p = 0.009, N = 13), and a strong negative correlation between AY errors and RT’s (PD: r = - 0.775, p = 0.000, N = 19; Controls: r = -0.728, p = 0.005, N = 13). Slope comparison from the linear regression analysis for CE showed that the influence of RT’s on CE was not different between groups (F (1,30) = 0.017, p = 0.898), further suggesting similar speed vs accuracy tradeoffs. Nonetheless, results showed significant differences between lines’ interception in the Go/NoGo task (F (1,31) = 8.317, p = 0.007), confirming a significantly higher number of CE for the PD group as the RT’s decreases compared to controls (Fig 5A). No differences were found for the AY errors for slope comparison (F (1,28) = 0.286, p = 0.597) and line interception (F (1,29) = 0.002, p = 0.913) (Fig 5B).

Concerning the correlation with disease stage, we found a positive moderate to strong correlation between A-Cue errors (motor reactive type of premature responses) and the disease stage (r_s_ = 0,636, p = 0,0043). A positive moderate correlation was also found for the number of proactive BX errors (r_s_ = 0,472, p = 0,041). Moreover, the only behavioral measure that showed any correlation with the total LEDd was the A-Cue errors (r_s_ = 0,652, p <0,003), suggesting a relation between medication dosage and premature responses.

## Discussion

In this study we have addressed the behavioral correlates of reduced impulse control in a medicated PD sample, simultaneously across 3 domains: cognitive, motor reactive and proactive impulsivity. Previous behavioral studies suggested isolated deficits in response inhibition in PD, in particular in the cases formally diagnosed as clinically established overt impulse control disorders. It is however important to consider subclinical multidomain manifestations, simultaneously across all impulsivity domains. This was the goal of the current study, which combined stop-signal and Go/no-Go tasks, with tasks requiring proactive inhibition, as well as control of cognitive impulsivity (the BART assessment task). We found important effects across all domains, when considering level of impairment, response strategies and correlation with disease stage.

It is now well recognized that ICD in late stage PD may occur as a consequence of DRT [46,47]. A neurobiological basis for such deficits is suggested by [11C] raclopride Positron Emission Tomography (PET) displacement studies which have demonstrated that PD patients with ICD exhibit task-related reduced binding in the ventral striatum, indicative of higher endogenous dopamine release. This may occur during gambling tasks or presentation of reward-related visual cues, as compared with PD patients without ICD [47,48]. Regarding the role of DRT, and in particular dopamine agonists, it has been suggested that they might impact on the processing of negative feedback during reward based learning [49]. Unexpected rewarding stimuli produce reinforcement by phasic increase in dopamine transmission that activates the D1 receptors in the direct pathway. Negative outcomes lead to a phasic decrease in dopamine transmission related to D2 activation in the indirect pathway.

However studies in PD in the absence of clinically defined impulse control disorder are lacking and even the ones with late stage ICD [49,50] lack simultaneous assessment of all impulsivity domains. Personality traits based scales [51] such as the Urgency-Premeditation-Perseverance-Sensation seeking Impulsive Behaviour Scale, are clinically useful, but do not address the neurobehavioural dimensions investigated in our study.

### Evidence for multidomain impairment in medicated PD patients and the relevance of neurobehavioral constructs

When studying the multidimensional nature of impulsivity deficits in PD taking dopaminergic medication, our results suggest distinct patterns across domains. The effects demonstrated for both motor impulsivity tasks could not be explained by differences in speed vs. accuracy tradeoffs (SAT) given that the analysis of RTs do not suggest differences in this domain. In fact, the evidence of a similar SAT slope regarding motor impulsivity, both validates our paradigm and show that different response strategies are not masking between group effects. Therefore, concerning motor reactivity, PD patients committed more errors as compared to the control group, irrespective of response strategy. Moreover, the results from the interception of the regression line between PD and controls further confirms the observed group difference (the number of reactive errors made by the PD group would always be greater as the RT diminishes in parallel for both groups). The notion that medicated PD patients might be impaired in reactive inhibitory tasks [30,32,51,52] then raises the question whether reactive deficits may mask proactive deficits if too many premature responses or omissions occur.

Available explanatory frameworks suggest that the loss of dopaminergic neurons eventually leads to functional changes in corticostriatothalamic circuits related to motor control, but likely also in other domains. One study by Giorgiev, Dirnberger, Wilkinson, Limousin and Jahanshahi [53] suggested that both STN-DBS patients and unoperated PD patients sustained difficulties in withholding a prepotent response when compared to controls. They further collected evidence that the stimulation per se has also a detrimental effect in this domain, particularly, when the no-go stimuli was frequent and when task difficulty was higher. Hershey et. al [54] suggested that ventral subthalamic stimulation affected the performance of PD patients in a go-nogo task. Cortical-STN connections constitute the hyperdirect pathway [54,55]. The STN provides excitatory commands to the globus pallidus interna, increasing the inhibition of thalamocortical transmission, and therefore inhibiting not just the primary motor cortex but also other cortical regions involved in other cognitive functions [13,56].

Given the notion that dopaminergic activity modulates impulsive behavior [3,18,26,57] and that our participants were assessed in “on” state medication, future studies should address the role of disease stage (which we found to be correlated with premature responses and proactive errors) and presence vs. absence of medication. Importantly, we aimed to understand whether the pattern of deficits goes beyond the motor impulsivity domain, or whether it also involves other domains, such as decision-making involving risk taking [46,58,59].

Regarding proactive impulsivity, we found a significant increase in omission errors for PD patients, a strategy that might led to the relative preservation of commission errors in this task. It is suggested that, contrary to the reactive domain, proactive impulsivity involves striatal outputs to the globus pallidus externa and then to globus pallidus interna, mediated by the STN [13]. In other words, particular STN neurons might be recruited during the commands sent to the GPi, specifically tuned to selective inhibition, comparing to the cortico-STN-GPi pathway, recruited during reactive inhibition [13]. Neurophysiological recordings in patients submitted to DBS also points to the role of the STN in evaluating contextual cues and their behavioral value [60]. Further evidence of a greater participation of the striatum–GPe–GPi pathway in tasks where the need to be stopped can be anticipated can be found in the literature [13,61,62]. One study using an fMRI paradigm with healthy subjects, suggested that the supplementary motor area, inferior frontal cortex and the STS as core regions involved in motor proactive adjustments [63]. The implication of the STS alongside with the frontal cortical regions like the inferior frontal cortex was also corroborated by additional work [64]. Like previously mentioned, the differences found for the number of omissions were specifically encountered for the AX-CPT task, which suggests a distinct strategy when proactive demands are present. We suggest that the cautious response style for this task could be secondary to the challenge imposed by the proactive condition. This idea is also corroborated by the positive correlation found specifically for the proactive BX errors and the disease stage. A study performed with PD patients submitted to DBS, suggested worse performance in PD patients in terms of proactive adjustment relative to controls while they were “on” medication state and off DBS stimulation [65]. Further evidence from PD patients submitted to subthalamotomy, showing poor proactive inhibitory control also highlights the importance of STN-cortical interactions on impulse control disorders in this population [66]. It has also been suggested that although dopamine medication reduces akinesia, it does not re-establish a normal pattern of internal control of motor response [67]. The interpretation is somewhat difficult because of the sparsity of studies using proactive task models for studying impulsivity in this population. Moreover, the ones that did, often relied on different methodology, often not studying proactive stopping per se, but other aspects of impulsive behavior. For example, in Bokura et. al [27] data from a computerized task was used as a way to assess the inhibitory function in general and the focus was placed in using event-related brain response correlates of inhibitory deficits in PD. Also, in Obeso and colleagues [28] although a conditional stop signal reaction time task was implemented, data analysis and theoretical considerations did not directly address proactive impulsivity. The conditional stop signal reaction time task is another task by which proactive interference might be studied (see [68] and [28]). Contrary to the AX-CPT used in our study, the main dependent variable for this task was an RT measure which takes into account the relative preparation cost calculation which reduces the weight of a bias in RT caused by the movement disorder per se. More studies regarding this impulsivity domain are needed, in particular using comprehensive studies like done here, which considered simultaneous multidomain deficits and response strategies.

We also found significant differences in the cognitive domain of impulsivity in our PD sample, namely in the response to punition. Previous findings suggested the involvement of a mesolimbic-frontal pathway in risk-taking tasks [69–71], where dopamine connects with mesolimbic regions (including striatum and midbrain) and frontal cortex (reciprocally) for stimulus salience and reward processing [70]. Therefore, insensitivity towards either reward and punishment are identified in this population [22,23,72], and suggested to be associated with the effects of dopamine depletion in frontal lobe regions, including fronto-striatal loops, basal ganglia projections for limbic structures, and orbitofrontal and dorsolateral prefrontal cortices [21,22]. Also, evidences with PD patients with clinically overt ICD showed a reduced ventral striatum activation during risk taking tasks [72]. Regarding this last structure, in the study of Torta et al. [25]) the authors found that the detrimental effect of DRT on a risk taking test is congruent with the overdose hypothesis which states that the ventral striatum receives more dopamine than needed (while dopamine levels are normalized in the dorsal striatum). The resulting impairment in its connection with the prefrontal cortex, might promote impulsive behaviors seen in risk taking tasks.

Concerning the limitations of this study, we did not test the PD patients in the off medication status. This would help disentangle if the proposed differences found in PD are a consequence of the medication per se or if it’s rather a consequence of the disease itself. As stated above, several studies suggest that dopaminergic medication, mainly dopamine agonists, have a detrimental effect on impulsivity [3,18,26,59]. However, these findings are still controversial [68], [73]. In our study we only found a correlation between dosage and premature A-cue response rates.

It would also be interesting to address the effects of DBS on impulsivity. Available studies also show some heterogeneity. For example, the study of Hershey et al. [74] suggests that in what concerns the inhibition of an ongoing voluntary motor behavior, assessed by a go/nogo task, patients on STN-DBS stimulation performed worse when compared to off DBS condition. Also in the study of Ballanger et al’s [75], STN-DBS was associated with an increase in CE errors accompanied by changes in cortical networks implicated in reactive and proactive response inhibition. In turn, van den Wildenberg et al [76] suggested a better performance in STN-DBS patients in a go/nogo task while on stimulation as compared to the off stimulation session. Evans et. al [73] found an association between STN-DBS with an increase of risky choices in a gambling task, but not with reward processing. Therefore, the use of a comprehensive approach on impulsivity, like the one used in our study, with patients submitted to DBS would also be useful to achieve a better comprehension of how different patterns of impulsive behavior are generated and maintained in this disease.

In sum, we found evidence for multidomain disruption in particular of motor and cognitive impulsivity in PD patients medicated with dopaminergic drugs, mainly at the cost of commission errors for the former, and insensitivity towards punition for the latter. Concerning proactive errors, they were correlated with disease stage, and moderated by response strategies (increased number of omissions). We propose that in medicated PD patients the nature of impairment of impulse control is multidimensional, suggesting that further studies should explore the particular role of each type of dopamine replacement drug on these dimensions.

## Supporting information

S1 Table Descriptive clinical information of each PD patient assessed. N–Patient number; UPDRS–Unified Parkinson’s Disease Rating Scale, H&Y–Hoehn and Yahr; LED–daily Levodopa Equivalent Dose; DA–Dopamine agonists; IR–Immediate Release; IC–Controlled Release. *LED calculations performed according to Tomlinson, C. L., et al. (2010), Systematic review of levodopa dose equivalency reporting in Parkinson's disease. Mov. Disord., 25: 2649–2653.
(DOCX)

## References

[1] Halliday G, Hely M, Reid W, Morris J. The progression of pathology in longitudinally followed patients with Parkinson’s disease. Acta Neuropathol. 2008;115(4):409–15. 10.1007/s00401-008-0344-8 18231798

[2] Velakoulis D., Lloyd J. Dementia with Lewy Bodies and Parkinson’s Disease In: O’Brien J., D. Ames & AB, editor. Dementia. 2nd ed New York: Arnold; 2000.

[3] Voon V, Gao J, Brezing C, Symmonds M, Ekanayake V, Fernandez H, et al Dopamine agonists and risk: impulse control disorders in Parkinson’s; disease. Brain. 2011;134(5):1438–46. 21596771 10.1093/brain/awr080 PMC3097893

[4] Cilia R, van Eimeren T. Impulse control disorders in Parkinson’s disease: seeking a roadmap toward a better understanding. Brain Struct Funct. 2011;216(4):289–99. 10.1007/s00429-011-0314-0 21541715 PMC3197927

[5] Isaias IU, Siri C, Cilia R, De Gaspari D, Pezzoli G, Antonini A. The relationship between impulsivity and impulse control disorders in Parkinson’s disease. Mov Disord. 2008;23(3):411–5. 10.1002/mds.21872 18067187

[6] Ribeiro M, Violante I, Bernardino I, Edden R, Castelo-Branco M. Abnormal relationship between GABA, neurophysiology and impulsive behavior in neurofibromatosis type 1. Cortex. 2015;64:194–208. 10.1016/j.cortex.2014.10.019 25437375 PMC4777301

[7] Everden J. Varieties of impulsivity. Psychopharmacology (Berl). 1999;146(4):348–61. 10550486 10.1007/pl00005481

[8] Dalley JW, Everitt BJ, Robbins TW. Impulsivity, Compulsivity, and Top-Down Cognitive Control. Neuron. 2011;69(4):680–94. 10.1016/j.neuron.2011.01.020 21338879

[9] World Health Organization. The ICD-10 Classification of Mental and Behavioural Disorders. Int Classif. 1992;10:1–267.

[10] Bechara A, Damasio H, Damasio AR. Emotion, decision making and the orbitofrontal cortex. Cereb Cortex. 2000;10(3):295–307. 10731224 10.1093/cercor/10.3.295

[11] Antonelli F, Ray N, Strafella AP. Impulsivity and Parkinson’s disease: More than just disinhibition. J Neurol Sci. 2011;310(1–2):202–7. 10.1016/j.jns.2011.06.006 21683964 PMC3440306

[12] van Belle J, Vink M, Durston S, Zandbelt BB. Common and unique neural networks for proactive and reactive response inhibition revealed by independent component analysis of functional MRI data. Neuroimage. 2014;103:65–74. 10.1016/j.neuroimage.2014.09.014 25224995

[13] Aron A. From Reactive to Proactive and Selective Control: Developing a Richer Model for Stopping Inappropriate Responses. Biol Psychiatry. 2011;69(12):e55–68. 10.1016/j.biopsych.2010.07.024 20932513 PMC3039712

[14] Rush B, Barch D, Braver T. Accounting for Cognitive Aging: Context Processing, Inhibition or Processing Speed? Aging, Neuropsychol Cogn. 2006;13(3–4):588–610. 10.1080/13825580600680703 16887791

[15] Voon V, Hassan K, Zurowski M, Souza M De, Thomsen T, Fox S, et al Prevalence of repetitive and reward-seeking behaviors in Parkinson disease. Neurology. 2006;67(7):1254–7. 10.1212/01.wnl.0000238503.20816.13 16957130

[16] Evans AH, Strafella AP, Weintraub D, Stacy M. Impulsive and compulsive behaviors in Parkinson’s disease. Mov Disord. 2009;24(11):1561–70. 10.1002/mds.22505 19526584

[17] Voon V, Fernagut P-O, Wickens J, Baunez C, Rodriguez M, Pavon N, et al Chronic dopaminergic stimulation in Parkinson’s disease: from dyskinesias to impulse control disorders. Lancet Neurol. 2009;8(12):1140–9. 10.1016/S1474-4422(09)70287-X 19909912

[18] Weintraub D, Siderowf AD, Potenza MN, Goveas J, Morales KH, Duda JE, et al Association of dopamine agonist use with impulse control disorders in Parkinson disease. Arch Neurol. 2006;63(7):969–73. 10.1001/archneur.63.7.969 16831966 PMC1761054

[19] Banca P, Voon V, Vestergaard MD, Philipiak G, Almeida I, Pocinho F, et al Imbalance in habitual versus goal directed neural systems during symptom provocation in obsessive-compulsive disorder. Brain. 2015;138(3):798–811. 25567322 10.1093/brain/awu379 PMC4339772

[20] Bodi N, Keri S, Nagy H, Moustafa a., Myers CE, Daw N, et al Reward-learning and the novelty-seeking personality: a between- and within-subjects study of the effects of dopamine agonists on young Parkinson’s patients. Brain. 2009;132(9):2385–95. 19416950 10.1093/brain/awp094 PMC2766178

[21] Brand M, Labudda K, Kalbe E. Decision-making impairments in patients with Parkinson ‘ s disease. Behav Neurol. 2004;15(3–4):77–85. 10.1155/2004/578354 15706051 PMC5488616

[22] Kobayakawa M, Koyama S, Mimura M, Kawamura M. Decision making in Parkinson’s disease: Analysis of behavioral and physiological patterns in the Iowa gambling task. Mov Disord. 2008;23(4):547–52. 10.1002/mds.21865 18069681

[23] Labudda K, Brand M, Mertens M, Ollech I, Markowitsch HJ, Woermann FG. Decision making under risk condition in patients with Parkinson’s disease: a behavioural and fMRI study. Behav Neurol. 2010;23(3):131–43. 10.3233/BEN-2010-0277 21098967 PMC5434409

[24] Simioni AC, Dagher A, Fellows LK. Dissecting the Effects of Disease and Treatment on Impulsivity in Parkinson’s Disease. J Int Neuropsychol Soc. 2012;18(6):942–51. 10.1017/S135561771200094X 23079116

[25] Torta DME, Castelli L, Zibetti M, Lopiano L, Geminiani G. On the role of dopamine replacement therapy in decision-making, working memory, and reward in Parkinson’s disease: Does the therapy-dose matter? Brain Cogn. 2009;71(2):84–91. 10.1016/j.bandc.2009.04.003 19442427

[26] van Eimeren T, Ballanger B, Pellecchia G, Miyasaki JM, Lang AE, Strafella AP. Dopamine Agonists Diminish Value Sensitivity of the Orbitofrontal Cortex: A Trigger for Pathological Gambling in Parkinson’s Disease? Neuropsychopharmacology. 2009;34(13):2758–66. 10.1038/sj.npp.npp2009124 19741594 PMC2972251

[27] Bokura H, Yamaguchi S, Kobayashi S. Event-related potentials for response inhibition in Parkinson’s disease. Neuropsychologia. 2005;43(6):967–75. 10.1016/j.neuropsychologia.2004.08.010 15716167

[28] Obeso I, Wilkinson L, Casabona E, Bringas ML, Álvarez M, Álvarez L, et al Deficits in inhibitory control and conflict resolution on cognitive and motor tasks in Parkinson’s disease. Exp Brain Res. 2011;212(3):371–84. 10.1007/s00221-011-2736-6 21643718

[29] Sinha N, Manohar S, Husain M. Impulsivity and apathy in Parkinson’s disease. J Neuropsychol. 2013;7(2):255–83. 10.1111/jnp.12013 23621377 PMC3836240

[30] Antonelli F, Strafella AP. Behavioral disorders in Parkinson’s disease: The role of dopamine. Parkinsonism Relat Disord. 2014;20:S10–2. 10.1016/S1353-8020(13)70005-1 24262157

[31] Voon V, Sohr M, Lang AE, Potenza MN, Siderowf AD, Whetteckey J, et al Impulse control disorders in Parkinson disease: a multicenter case—control study. Ann Neurol. 2011;69(6):986–96. 10.1002/ana.22356 21416496

[32] Gauggel S, Rieger M, Feghoff T. Inhibition of ongoing responses in patients with Parkinson’s disease. J Neurol Neurosurg Psychiatry. 2004;75:539–44. 15026491 10.1136/jnnp.2003.016469 PMC1739013

[33] Nombela C, Rittman T, Robbins TW, Rowe JB. Multiple Modes of Impulsivity in Parkinson’s Disease. PLoS One. 2014;9(1):85747. 10.1371/journal.pone.0085747 PMC3897514 24465678

[34] Antonelli F, Ko JH, Miyasaki J, Lang AE, Houle S, Valzania F, et al Dopamine-agonists and impulsivity in Parkinson’s disease: Impulsive choices vs. impulsive actions. Hum Brain Mapp. 2014;35(6):2499–506. 10.1002/hbm.22344 24038587 PMC4452224

[35] Tomlinson CL, Stowe R, Patel S, Rick C, Gray R, Clarke CE. Systematic review of levodopa dose equivalency reporting in Parkinson’s disease. Mov Disord. 2010;25(15):2649–53. 10.1002/mds.23429 21069833

[36] Beck AT, Steer RA, Brown GK. Manual for the Beck depression inventory-II. In: Manual for the Beck depression inventory—II. 1996 p. 1–82.

[37] Campos RC, Gonçalves B. The portuguese version of the Beck Depression Inventory-II (BDI-II): Preliminary psychometric data with two nonclinical samples. Eur J Psychol Assess. 2011;27(4):258–64.

[38] Caeiro L, Silva T, JM F, J P-R, Ml F. Metric properties of the portuguese version of the apathy evaluation scale. Psicol Saúde Doenças. 2012;13(2):266–82.

[39] Freitas S, Simões MR, Alves L, Santana I. Montreal Cognitive Assessment (MoCA): Normative study for the Portuguese population. J Clin Exp Neuropsychol. 2011;33(9):989–96. 10.1080/13803395.2011.589374 22082082

[40] Hughes AJ, Daniel SE, Kilford L, Lees AJ. Accuracy of clinical diagnosis of idiopathic Parkinson ‘ s disease: a clinico-pathological study of 100 cases. 1992;181–4. 10.1136/jnnp.55.3.181 PMC1014720 1564476

[41] Dockree PM, Kelly SP, Robertson IH, Reilly RB, Foxe JJ. Neurophysiological markers of alert responding during goal-directed behavior: A high-density electrical mapping study. Neuroimage. 2005;27(3):587–601. 10.1016/j.neuroimage.2005.05.044 16024257

[42] Brainard D. The Psychophysics Toolbox. Spat Vis. 1997;10(4):433–6. 9176952

[43] Lejuez CW, Read JP, Kahler CW, Richards JB, Ramsey SE, Stuart GL, et al Evaluation of a behavioral measure of risk taking: The Balloon Analogue Risk Task (BART). J Exp Psychol Appl. 2002;8(2):75–84. 12075692 10.1037//1076-898x.8.2.75

[44] Scheiner SM. MANOVA: Multiple response variables and multispecies interactions In: Scheiner S, Gurevitch J., editors. Design and analysis of ecological experiments. 2nd ed Oxford, England: Oxford University Press; 2001.

[45] Gaus W, Mayer B, Muche R. Interpretation of Statistical Significance—Exploratory Versus Confirmative Testing in Clinical Trials, Epidemiological Studies, Meta-Analyses and Toxicological Screening (Using Ginkgo biloba as an Example). Clin Exp Pharmacol. 2015;5(4).

[46] Wu K, Politis M, Piccini P. Parkinson disease and impulse control disorders: a review of clinical features, pathophysiology and management. Postgrad Med J. 2009;85(1009):590–6. 10.1136/pgmj.2008.075820 19892894

[47] Weintraub D, Koester J, Potenza M, Siderowf AD, Stacy M, Voon V, et al Impulse Control Disorders in Parkinson Disease: a cross-sectional study of 3090 patients. Arch Neurol. 2010;67(5):589–95. 10.1001/archneurol.2010.65 20457959

[48] O’Sullivan SS, Wu K, Politis M, Lawrence a. D, Evans a. H, Bose SK, et al Cue-induced striatal dopamine release in Parkinson’s disease-associated impulsive-compulsive behaviours. Brain. 2011;134(4):969–78. 21349901 10.1093/brain/awr003

[49] Frank MJ, Seeberger LC, O’Reilly RC. By Carrot or by Stick: Cognitive Reinforcement Learning in Parkinsonism. Science. 2004;306(5703):1940–3. 10.1126/science.1102941 15528409

[50] Vela L, Martínez Castrillo JC, García Ruiz P, Gasca-Salas C, Macías Macías Y, Pérez Fernández E, et al The high prevalence of impulse control behaviors in patients with early-onset Parkinson’s disease: A cross-sectional multicenter study. J Neurol Sci. 2016;368:150–4. 10.1016/j.jns.2016.07.003 27538621

[51] Bayard S, Joly E, Ghisletta P, Rossignol A, Herades Y, Geny C, et al A multidimensional approach to impulsivity in Parkinson’s disease: measurement and structural invariance of the UPPS Impulsive Behaviour Scale. Psychol Med. 2016;46(14):2931–41. 10.1017/S0033291716001586 27460484

[52] Hart RP, Wade JB, Calabrese VP, Colenda CC. Vigilance Performance in Parkinson ‘s Disease and Depression. J Clin Exp Neuropsychol. 1998;20(1):111–7. 10.1076/1380-3395(199802)20:1;1-P;FT111 9672825

[53] Georgiev D, Dirnberger G, Wilkinson L, Limousin P, Jahanshahi M. In Parkinson’s disease on a probabilistic Go/NoGo task deep brain stimulation of the subthalamic nucleus only interferes with withholding of the most prepotent responses. Exp Brain Res. 2016;234(4):1133–43. 10.1007/s00221-015-4531-2 26758720 PMC4785203

[54] Hershey T, Campbell MC, Videen TO, Lugar HM, Weaver PM, Hartlein J, et al Mapping Go-No-Go performance within the subthalamic nucleus region. Brain. 2010;133(12):3625–34. 20855421 10.1093/brain/awq256 PMC2995882

[55] Magill PJ, Sharott A, Bevan MD, Brown P, Bolam JP. Synchronous unit activity and local field potentials evoked in the subthalamic nucleus by cortical stimulation. J Neurophysiol. 2004;92(2):700–14. 10.1152/jn.00134.2004 15044518

[56] Nambu A, Tokuno H, Takada M. Functional significance of the cortico-subthalamo-pallidal “hyperdirect” pathway. Neurosci Res. 2002;43(2):111–7. 12067746 10.1016/s0168-0102(02)00027-5

[57] Wylie SA, van Wouwe NC, Godfrey SG, Bissett PG, Logan GD, Kanoff KE, et al Dopaminergic medication shifts the balance between going and stopping in Parkinson’s disease. Neuropsychologia. 2018;109(December 2017):262–9. 10.1016/j.neuropsychologia.2017.12.032 29269306

[58] Zheng D, Oka T, Bokura H, Yamaguchi S. The key locus of common response inhibition network for no-go and stop signals. J Cogn Neurosci. 2008;20(8):1434–42. 10.1162/jocn.2008.20100 18303978

[59] Claassen DO, van den Wildenberg WPM, Ridderinkhof KR, Jessup CK, Harrison MB, Wooten GF, et al The risky Business of Dopamine Agonists in Parkinson Disease and Impulse Control Disorders. Behav Neurosci. 2011;125(4):492–500. 10.1037/a0023795 21604834 PMC3144294

[60] Kühn AA, Williams D, Kupsch A, Limousin P, Hariz M, Schneider GH, et al Event-related beta desynchronization in human subthalamic nucleus correlates with motor performance. Brain. 2004;127(4):735–46. 14960502 10.1093/brain/awh106

[61] Vink M, Kahn RS, Raemaekers M, van den Heuvel M, Boersma M, Ramsey NF. Function of striatum beyond inhibition and execution of motor responses. Hum Brain Mapp. 2005;25(3):336–44. 10.1002/hbm.20111 15852388 PMC6871687

[62] Wylie S, Claassen D, Huizenga H, Schewel K, Ridderinkhof K, Bashore T, et al Dopamine Agonists and the Supression of Impulsive Motor Actions in Parkinson’s Disease. J Cogn Neurosci. 2012;24(8):1709–24. 10.1162/jocn_a_00241 22571461 PMC3657467

[63] Aron AR, Behrens TE, Smith S, Frank MJ, Poldrack RA. Triangulating a Cognitive Control Network Using Diffusion-Weighted Magnetic Resonance Imaging (MRI) and Functional MRI. J Neurosci. 2007;27(14):3743–52. 10.1523/JNEUROSCI.0519-07.2007 17409238 PMC6672420

[64] Jahfari S, Stinear CM, Claffey M, Verbruggen F, Aron AR. Responding with Restraint: What Are the Neurocognitive Mechanisms? J Cogn Neurosci. 2009;22(7):1479–92. 10.1162/jocn.2009.21307 PMC2952035 19583473

[65] Obeso I, Wilkinson L, Rodríguez-Oroz MC, Obeso JA, Jahanshahi M. Bilateral stimulation of the subthalamic nucleus has differential effects on reactive and proactive inhibition and conflict-induced slowing in Parkinson’s disease. Exp Brain Res. 2013;226(3):451–62. 10.1007/s00221-013-3457-9 23525560

[66] Obeso I, Wilkinson L, Casabona E, Speekenbrink M, Bringas ML, Álvarez M, et al The subthalamic nucleus and inhibitory control: Impact of subthalamotomy in Parkinson’s disease. Brain. 2014;137(5):1470–80. 24657985 10.1093/brain/awu058

[67] Favre E, Ballanger B, Thobois S, Broussolle E, Boulinguez P. Deep Brain Stimulation of the Subthalamic Nucleus, but not Dopaminergic Medication, Improves Proactive Inhibitory Control of Movement Initiation in Parkinson’s Disease. Neurotherapeutics. 2013;10(1):154–67. 10.1007/s13311-012-0166-1 23184315 PMC3557357

[68] Obeso I, Wilkinson L, Jahanshahi M. Levodopa medication does not influence motor inhibition or conflict resolution in a conditional stop-signal task in Parkinson’s disease. Exp Brain Res. 2011;213(4):435–45. 10.1007/s00221-011-2793-x 21796541

[69] Perez-Lloret S, Rascol O. Dopamine receptor agonists for the treatment of early or advanced Parkinsons disease. CNS Drugs. 2010;24(11):941–68. 10.2165/11537810-000000000-00000 20932066

[70] Rao H, Korczykowski M, Pluta J, Hoang A, Detre J a. Neural correlates of voluntary and involuntary risk taking in the human brain: An fMRI Study of the Balloon Analog Risk Task (BART). Neuroimage. 2008;42(2):902–10. 10.1016/j.neuroimage.2008.05.046 18582578 PMC9475445

[71] Tom SM, Fox CR, Trepel C, Poldrack RA. The Neural Basis of Loss Aversion in Decision Making Under Risk. Science. 2007;315(5811):515–518. 10.1126/science.1134239 17255512

[72] Rao H, Mamikonyan E, Detre JA, Siderowf AD, Stern MB, Potenza MN, et al Decreased ventral striatal activity with impulse control disorders in Parkinson’s Disease. Mov Disord. 2010;25(11):1660–9. 10.1002/mds.23147 20589879 PMC3063061

[73] Evens R, Hoefler M, Biber K, Lueken U. The Iowa Gambling Task in Parkinson’s disease: A meta-analysis on effects of disease and medication. Neuropsychologia. 2016;91:163–72. 10.1016/j.neuropsychologia.2016.07.032 27475264

[74] Hershey T, Revilla FJ, Wernle A, Gibson PS, Dowling JL, Perlmutter JS. Stimulation of STN impairs aspects of cognitive control in PD. Neurology. 2004;62(7):1110–4. 15079009 10.1212/01.wnl.0000118202.19098.10

[75] Ballanger B, Van Eimeren T, Moro E, Lozano AM, Hamani C, Boulinguez P, et al Stimulation of the subthalamic nucleus and impulsivity: Release your horses. Ann Neurol. 2009;66(6):817–24. 10.1002/ana.21795 20035509 PMC2972250

[76] van den Wildenberg WPM, van Boxtel GJM, van der Molen MW, Bosch DA, Speelman JD, Brunia CHM. Stimulation of the Subthalamic Region Facilitates the Selection and Inhibition of Motor Responses in Parkinson ‘ s Disease. J Cogn Neurosci. 2006;18(4):626–36. 10.1162/jocn.2006.18.4.626 16768365

