# Supplementary material for: Impulsivity across reactive, proactive and cognitive domains in Parkinson's disease on dopaminergic medication: Evidence for multiple domain impairment
Source: PLoS One. 2019 Feb 13;14(2):e0210880. doi: 10.1371/journal.pone.0210880 (PMC6373905; doi:10.1371/journal.pone.0210880)
Supplement: S1 Table — N–Patient number; UPDRS–Unified Parkinson’s Disease Rating Scale, H&Y–Hoehn and Yahr; LED–daily Levodopa Equivalent Dose; DA–Dopamine agonists; IR–Immediate Release; IC–Controlled Release. *LED calculations performed according to Tomlinson, C. L., et al. (2010), Systematic review of levodopa dose equivalency reporting in Parkinson's disease. Mov. Disord., 25: 2649–2653. (DOCX) [file pone.0210880.s001.docx]

| N | Disease Duration | UPDRS ON | UPDRS OFF | H&Y | Medication | LED* DA | LED* Total | |
| --- | --- | --- | --- | --- | --- | --- | --- | --- |
| 1 | 3 | 19 | 33 | 2 | Levodopa+Carbidopa 750 mg IR, Ropinirole 2 mg | 40 | 790 | |
| 2 | 2 | 31 | 52 | 2 | Levodopa+Carbidopa 400 mg IR | 0 | 400 | |
| 3 | 2 | 26 | 40 | 2 | Levodopa+Carbidopa 400 mg IR | 0 | 400 | |
| 4 | 13 | 35 | 36 | 2 | Levodopa+Benserazide 600 mg IR, Ropinirole 4 mg | 80 | 680 | |
| 5 | 14 | 23 | 54 | 3 | Levodopa+Carbidopa 1225 mg IR, Ropinirole 12 mg, Amantadine 200 mg, Selegiline 10 mg | 540 | 1765 | |
| 6 | 5 | 32 | 47 | 2 | Levodopa+Carbidopa 1000 mg IR, Ropinirole 2 mg, Amantadine 100 mg | 140 | 1140 |  |
| 7 | 8 | 26 | 62 | 3 | Levodopa+Carbidopa 500mg IR + 200mg CR , Ropinirole 16 mg, Rasagiline 1 mg | 420 | 1070 | |
| 8 | 5 | 28 | 39 | 2 | Levodopa+Carbidopa 300 mg IR | 0 | 300 | |
| 9 | 3 | 33 | 48 | 3 | Levodopa+Carbidopa 400 mg IR, Pramipexole 0.26 mg | 26 | 426 | |
| 10 | 5 | 30 | 44 | 2 | Levodopa+Carbidopa 1125 mg IR + 200 mg CR, Ropinirole 4 mg, Rasagiline 1 mg | 180 | 1455 | |
| 11 | 3 | 3 | 38 | 2.5 | Levodopa+Carbidopa 500 mg IR + 200 mg CR, Entacapone 165 mg , Ropinirole 16 mg, Amantadine 200 mg, Rasagiline 1 mg | 785 | 1435 | |
| 12 | 10 | 35 | 41 | 3 | Levodopa+Carbidopa 750 mg IR + 200 mg CR, Ropinirole 12 mg | 240 | 1140 | |
| 13 | 13 | 23 | 32 | 2 | Levodopa+Carbidopa 300 mg IR | 0 | 300 | |
| 14 | 3 | 23 | 26 | 2 | Levodopa+Carbidopa 300 mg IR | 0 | 300 | |
| 15 | 11 | 17 | 27 | 2.5 | Levodopa+Carbidopa 200 mg IR, Ropinirole 8 mg, Selegiline 10 mg | 260 | 460 | |
| 16 | 2 | 35 | 44 | 2.5 | Levodopa+Carbidopa 400 mg IR | 0 | 400 | |
| 17 | 4 | 28 | 38 | 2 | Levodopa+Carbidopa 550 mg IR | 0 | 550 | |
| 18 | 5 | 25 | 43 | 2.5 | Levodopa+Carbidopa 400 mg IR | 0 | 400 | |
| 19 | 14 | 8 | 46 | 2 | Levodopa+Carbidopa 550 mg IR + 200 mg CR, Ropinirole 10 mg, Amantadine 200 mg, Rasagiline 1 mg | 500 | 1200 | |
| 20 | 4 | 17 | 29 | 2 | Levodopa+Carbidopa 300 mg IR | 0 | 300 | |
| 21 | 21 | 25 | 46 | 2 | Levodopa+Carbidopa 550 mg IR + 200 mg CR | 0 | 700 | |
